# Bioinformatic prediction of transcription factor binding sites at promoter regions of genes for photoperiod and vernalization responses in model and temperate cereal plants

**DOI:** 10.1186/s12864-016-2916-7

**Published:** 2016-08-08

**Authors:** Fred Y. Peng, Zhiqiu Hu, Rong-Cai Yang

**Affiliations:** 1 Feed Crops Section, Alberta Agriculture and Forestry, 7000 - 113 Street, Edmonton, AB T6H 5T6 Canada; 2 Department of Agricultural, Food and Nutritional Science, University of Alberta, 410 Agriculture/Forestry Centre, Edmonton, AB T6G 2P5 Canada

**Keywords:** Cereal plants, Photoperiod, Position weight matrices, Transcription factor binding sites, Transcription regulation, Vernalization, Flowering genes

## Abstract

**Background:**

Many genes involved in responses to photoperiod and vernalization have been characterized or predicted in Arabidopsis (*Arabidopsis thaliana*), Brachypodium (*Brachypodium distachyon*), wheat (*Triticum aestivum*) and barley (*Hordeum vulgare*). However, little is known about the transcription regulation of these genes, especially in the large, complex genomes of wheat and barley.

**Results:**

We identified 68, 60, 195 and 61 genes that are known or postulated to control pathways of photoperiod (PH), vernalization (VE) and pathway integration (PI) in Arabidopsis, Brachypodium, wheat and barley for predicting transcription factor binding sites (TFBSs) in the promoters of these genes using the FIMO motif search tool of the MEME Suite. The initial predicted TFBSs were filtered to confirm the final numbers of predicted TFBSs to be 1066, 1379, 1528, and 789 in Arabidopsis, Brachypodium, wheat and barley, respectively. These TFBSs were mapped onto the PH, VE and PI pathways to infer about the regulation of gene expression in Arabidopsis and cereal species. The GC contents in promoters, untranslated regions (UTRs), coding sequences and introns were higher in the three cereal species than those in Arabidopsis. The predicted TFBSs were most abundant for two transcription factor (TF) families: MADS-box and CSD (cold shock domain). The analysis of publicly available gene expression data showed that genes with similar numbers of MADS-box and CSD TFBSs exhibited similar expression patterns across several different tissues and developmental stages. The intra-specific Tajima *D*-statistics of TFBS motif diversity showed different binding specificity among different TF families. The inter-specific Tajima *D*-statistics suggested faster TFBS divergence in TFBSs than in coding sequences and introns. Mapping TFBSs onto the PH, VE and PI pathways showed the predominance of MADS-box and CSD TFBSs in most genes of the four species, and the difference in the pathway regulations between Arabidopsis and the three cereal species.

**Conclusion:**

Our approach to associating the key flowering genes with their potential TFs through prediction of putative TFBSs provides a framework to explore regulatory mechanisms of photoperiod and vernalization responses in flowering plants. The predicted TFBSs in the promoters of the flowering genes provide a basis for molecular characterization of transcription regulation in the large, complex genomes of important crop species, wheat and barley.

**Electronic supplementary material:**

The online version of this article (doi:10.1186/s12864-016-2916-7) contains supplementary material, which is available to authorized users.

## Background

The genetic basis of flowering time control has been studied extensively in the model plant Arabidopsis (*Arabidopsis thaliana*) with over 200 putative flowering-related genes being identified [1, 2]. These genes have served as the reference for genome-wide prediction of flowering gene homologs in other plants including a cereal model species Brachypodium (*Brachypodium distachyon*), and two important cereal crops in short-season cropping regions, wheat (*Triticum aestivum*) and barley (*Hordeum vulgare*) [3, 4]. Such an approach assumes evolutionary conservation of flowering genes between Arabidopsis and other plants, but, different flowering pathways show varying degrees of evolutionary conservation between Arabidopsis and cereals [5–9]. The photoperiod pathway, particularly the circadian clock entrainment, is relatively conserved between Arabidopsis and monocot species [10]. For example, a recent study reported that two-thirds of the key circadian clock components are conserved in Arabidopsis and barley [11], including critical photoperiod genes like *CONSTANS* (*CO*), *EARLY FLOWERING 4* (*ELF4*), and *PSEUDO RESPONSE REGULATORs* (*PRRs*). In contrast, another major flowering-related pathway controlling the vernalization response pathway is reported to be less conserved between Arabidopsis and monocots [5–9].

Like in all other regulated genes, the expression of flowering genes is regulated by regions of non-coding DNA known as *cis*-regulatory elements (CREs) that contain transcription factor binding sites (TFBSs) to regulate the gene transcription. The two most well-characterized types of CREs are promoters and enhancers [12]. A promoter is about 100–1000 bp long and it is often located at upstream of a transcription start site (TSS) of a regulated gene. A specific DNA sequence in the promoter provides a secure initial binding site for RNA polymerase and for other transcription factors (TFs) that recruit RNA polymerase. Thus the positions and sequences of promoters can be inferred with relative ease from their immediate physical proximity to the regulated genes. On the other hand, while an enhancer is also a short (50–1500 bp) region of DNA that can be bound by TFs (i.e., activators) for transcription regulation of a gene, it is located up to 1 Mbp away from the regulated gene in either upstream or downstream from the TSSin the forward or backward direction. Thus there is currently no single ‘enhancer marker’ for genome-wide identification of enhancers because all conservation- or epigenomics-based predictions show that some enhancer regions are missed (false negatives), and other sequences predicted to be active enhancers cannot be validated by complementary methods (false positives).

Some empirical studies have revealed the effects of sequence variations surrounding TFBSs at promoter regions of the flowering-related genes on the photoperiod sensitivity and vernalization requirement in cereal species. A 2,089 bp deletion in the upstream of the wheat *PHOTOPERIOD1* (*PPD1*) gene (*Ppd-A1* and *Ppd-D1*) can reduce the photoperiod sensitivity, resulting in early heading time [13–15]. Similarly, an insertion of 308 bp and a deletion of 1,085 bp in the upstream region of *Ppd-A1* were shown to accelerate heading by 7 – 9 days compared with the photoperiod-sensitive genotype at this gene locus [16]. This photoperiod insensitive phenotype was caused by the removal of one or more regulatory regions, which are involved in TF binding and regulation of *PPD1* [14, 16]. Three independent deletions in the promter regions of *VERNALIZATION1* (*VRN1*) in *Triticum monococcum* caused elevated gene expression and reduced vernalization requirement [17]. Coversely, single-nucleotide polymorphisms (SNPs) in the promoter of *VRN1-D* and intron deletion resulted in its reduced expression and increased vernalization requirement in wheat [18]. Furthermore, insertion/deletion in the first intron of *VRN1* in wheat and barley can also reduce their vernalization requirement [19–21]. Recently, Kippes et al. [22] showed that three adajcent SNPs in a regulatory region of the wheat VRN-D4 first intron disrupt the binding of GLYCINE-RICH RNA-BINDING PROTEIN 2 (TaGRP2), a known repressor of *VRN1* expression. The effect of intron deletion is intriguing because introns have been historically considered as junk DNA, though recent work shows that the first intron can also habour regulatory signals [22, 23]. Further sequence analysis showed that variations causing changes in photoperiod sensitivity and vernalization requirement in wheat and barley were not detected in the protein-coding regions of *PPD1* or *VRN1* [16, 17]. Therefore, TFBS identification, like gene annotation, is also essential to elucidate the molecular basis of flowering transition in plant species.

Experimental techniques are available to identify TFBSs in the promoter regions of target genes, including CHIP-Seq (chromatin immunoprecipitation coupled with massively parallel DNA sequencing) and protein binding microarray (PBM) [24–26]. However, these techniques have limitations such as the GC-content bias and high cost, and require a considerable amount of downstream data processing. Thus, bioinformatic approaches have also been developed for TFBS prediction and they typically depend on position weight matrices (PWMs) corresponding to TFs as the scoring matrices [27–29]. Most PWMs are derived from binding motifs that are determined experimentally for a given TF, and they can be obtained in public databases such as JASPAR, TRANSFAC, and CIS-BP [24, 30, 31]. Additionally, approaches using GC content as DNA free energy profiles to predict TFBSs in plants have also been developed [32, 33].

To date, the TFBS annotation in plant regulatory sequences is still largely limited, with most work being done in model plant Arabidopsis, such as AGRIS [34]. The objective of this study was to predict distributions and related properties of TFBSs in the promoter regions of flowering-related genes in the Arabidopsis genome and the genomes of three cereal species, Brachypodium, wheat and barley. Model cereal plant Brachypodium is included as an effort to bridge the knowledge gap between the well-characterized Arabidopsis genome and the large, complex wheat/barley genomes. Despite the potential significance of enhancers and other CREs in controlling the level of transcription of flowering genes in response to environmental stimuli, this study focused on predicting TFBSs in the promoter regions of the genes only. Our approach consisted of the following steps. First, we identified the genes involved in the pathways of photoperiod (PH) and vernalization (VE), and the pathway integration (PI) genes that control the convergence point of the PH and VE pathways [6, 9, 35] in Arabidopsis, Brachypodium, wheat and barley. Second, we predicted TFBSs in the promoter regions of these genes and assessed the divergence of the TFBSs relative to their coding sequences (CDS) and introns of the orthologous genes. Third, we analyzed the public microarray data sets to assess the relationship between putative TFBSs and gene expression profiles. Finally, we mapped putative TFBSs onto the genes of the PH, VE and PI pathways in the four species, with the three genomes (A, B, and D) of allohexaploid wheat being treated separately.

## Methods

### Identification of genes in photoperiod, vernalization and pathway integration

In this study, we only focused on genes whose transcription regulation is related to the PH, VE and PI pathways. The lists of genes in Arabidopsis, wheat and barley were taken from our previous work [4], and their orthologous genes in Brachypodium were added, largely following Higgins et al. [3]. Because some gene identifiers reported in Higgins et al. [3] were no longer present in the latest release of the Brachypodium genome used in this analysis, we mapped their old IDs to the new IDs using their protein sequences with BLASTP [36]. If known genes in wheat and barley were available in GenBank, their corresponding genes in EnsemblPlants were identified using their protein sequences and BLASTP [36].

### Sequence retrieval

The promoter sequences of the PH, VE and PI genes in Arabidopsis, Brachypodium, wheat and barley were retrieved from the RSAT Plants server [37], using transcript as position reference. Here, we defined a promoter region as a stretch of up to 1000 bp upstream from the TSS of each gene. If another adjacent gene is located within less than 1000 bp upstream of the study gene, we only retrieved the longest possible promoter sequence to avoid any overlap with the upstream gene. The promoter sequences were examined to exclude those with more than 90 % N’s (representing gaps) in wheat and barley from further analyses. The coding and intron sequences of the genes were retrieved using the Ensembl Plants database [38], via its Perl API (application program interface).

### Prediction of transcription factor binding sites (TFBSs) in the promoter regions

To predict TFBSs in the upstream promoter regions, we installed a standalone version of the MEME suite, which includes the FIMO motif search tool [28, 39]. For this FIMO motif discovery, we first collected a non-redundant set of position-weight matrices (PWMs) for binding profiles of known TFs. Briefly, we downloaded 64 PWMs of plant transcription factors from JASPAR (http://jaspar.genereg.net) [30]. Then we combined a total of 725 PWMs of five species in CIS-BP (http://cisbp.ccbr.utoronto.ca) [24]: Arabidopsis (309), Brachypodium (192), maize (*Zea mays*; 209), wheat (9) and barley (6). The PWMs of another cereal crop maize were included as well because it has more than 200 PWMs. As these PWMs were from different databases and species, duplicated or very similar PWMs for a given TF were occasionally found. These redundant PWMs were removed through comparing (i) the motif ID with its corresponding TF name on the motif definition line, (ii) the matrices themselves. For example, only one PWM was retained among those with over 80 % similarity (including their reverse complement matrices), because a TF can generally tolerate a limited number of substitutions within its binding site [40]. We implemented this cleaning process in R [41]. Additionally, among duplicated or very similar PWMs, we retained the ones derived from experimental data. After these filtering steps, the resulting set of 371 unique PWM models were used for TFBS prediction, corresponding to 345 cognate TFs.

For the FIMO background model, we retrieved the promoter sequences of all annotated genes for each species (excluding the genes in this study) from RSAT, and generated 0-order Markov-chain frequencies of the four nucleotides for each species, with the fasta-get-Markov tool in the MEME suite (Additional file 1: Table S1). For other main options of FIMO, we searched TFBSs on either strand of the promoter sequences, and set Q-value threshold as 0.2 and ‘motif-pseudo’ as 10^-8^. The Q-value cutoff was set as suggested by Storey [42], to control false discovery rate (FDR). The value of the motif-pseudo parameter was added to avoid zero probability on any position in a matrix, and we found the FIMO default of 0.1 is too large for PWMs, as some positions in many PWMs contain values of far smaller than 0.1 (Data not shown). The FIMO default of 0.1 might be more appropriate for position frequency matrices or PFMs instead of PWMs. Further cleaning of the FIMO output was carried out as well. For putative binding sites of the same TF family predicted on overlapping promoter sequence regions (or start/end positions covered by a long TFBS of the same TF family), we only retained the longest one (which often covers several short motifs) or the one with the smallest *P*-value calculated by FIMO.

The TF information on each predicted TFBS was added using the TF-motif association files from CIS-BP for the following species: Arabidopsis, Brachypodium, maize, wheat, barley, *Antirrhinum majus*, *Petunia* x *hybrida*, and *Pisum sativum*. It should be noted that the PWMs were derived from all the eight species listed, not just the four species used for our study.

### Gene expression analysis

The Affymetrix microarray data in Arabidopsis, Brachypodium, wheat and barley were taken from the Plant Expression Database PLEXdb, with their experiment accession identifications being AT40, BD1, TA3 and BB3, respectively. However, the data set form Brachypodium (BD1) is a time-series (0 to 48 h) assay under four diurnal/circadian treatments: LLHH (Light day, Light night, Hot day, Hot night), LDHH (Light day, Dark night, Hot day, Hot night), LDHC (Light day, Dark night, Hot day, Cold night), and LLHC (Light day, Light night, Hot day, Cold night) (Mockler T. unpublished data).

From these microarray data sets, we only considered the tissues or developmental stages of high relavence to flowering, such as leaf and floral organs, but not those of little relavence to flowering such as root, shoot and seed (embryo and endosperm). The raw data files (.CEL) were normalized and transformed into log_2_-based expression values in a consistent procedure using Bioconductor packages in the R statistical environment [41, 43]. Heat maps of expression data were generated with the heatmap.2 function in the R gplots package.

It should be noted that RNA-seq data would be preferred given its advantages over the microarray data including unbiased detection of novel transcripts and increased specificity and sensitivity of detecting differential expression [44]. However, it was difficult to find RNA-seq data from the same or similar plant tissues of all four species for valid and reliable cross-species comparison. Therefore, our assessment of gene expression profiles was based on the microarray data that were publicably available for the similar plant issues of all four species.

### Statistical analysis of TFBS divergence

For this analysis, we first clustered all genes into ortholog groups (OG) with OrthoMCL and OrthoMCL-DB [45, 46]. The orthology relationship of the genes in the four species was then utilized to assess the interspecific divergence of predicted TFBSs, coding sequences and introns of the same genes (Additional file 2). The divergence analysis was based on Tajima’s *D* statistics as implemented in VariScan [47, 48]. For its input, multiple sequence alignment was performed for sequences in each OG with ClustalW2 [49].

The estimated intraspecific diversity of TFBSs may reflect the DNA-binding preference within TF families, whereas the estimated interspecific divergence may aid in our understanding the evolution of gene regulation. Thus, we analyzed the TFBS diversity of these genes within and between these four species using Tajima’s *D* statistics. A negative *D* value would be indicative of more conservative (or specific) binding sites than a zero or positive *D* value [47]. Thus the estimates of Tajima’s *D* statistics here simply served to summarize the predicted TFBS motif variation within and among the four plant species rather than demographic and evolutionary inferences as often intended in the use of Tajima’s *D* statistics.

### Mapping TFBSs onto flowering pathways

Given the obvious differences in flowering time regulation by PH, VE and PI pathways between Arabidopsis and the cereal plants (cf. Fig. 2 of [6]), we mapped the predicted TFBSs onto the appropriate pathways through tracking the regulated genes along the routes of the pathways. This mapping was done to infer about the species differences in promoter-driven regulation of gene expression for triggering flowering pathways and responding to environmental stimuli such as cold and long days. We used the Arabidopsis flowering pathways (WP622) in WikiPathways as a template and incorporated the known differences with the cereal species [5, 6, 8, 9] to produce a new set of pathway network charts using PathVisio (version 3.2.1), a pathway analysis tool [50, 51].

The homologous genes in the three cereal species were found using the orthology relationship (Additional file 2). Three separate pathway network charts were produced for the allohexaploid wheat, each representing one of the three homoeologs, A, B and D genomes (paralogs arising from polyploidy). By matching with the same flowering genes for TFBS prediction (see Additional file 3), we mapped our predicted TFBSs of the corresponding genes on the pathway network charts.

## Results

### Genes in photoperiod, vernalization and pathway integration and their promoter regions

The numbers of genes in PH, VE and PI and their promoter sequences are summarized in Table 1. A total of 68, 60, 195 and 61 genes were found in Arabidopsis, Brachypodium, wheat and barley, respectively, but not every gene had a promoter sequence. The lack of promoter sequences in the two Arabidopsis genes, one in PH and the other in VE, is due to their overlap with upstream adjacent genes. These overlaps are: a protein-coding gene AT2G18915 [*ADAGIO2* (*ADO2*)/*LOV KELCH PROTEIN 2* (*LKP2*)] on the reverse strand overlaps with a noncoding RNA gene AT2G18917 on the forward strand, whereas AT2G18880 [VERNALIZATION5/VIN3-LIKE 2 (*VEL2*)/VIN3-LIKE 3 (*VIL3*)] overlaps with AT2G18876 encoding a microtubule-associated protein; both genes are on the forward strand. The promoter sequences of 32 wheat genes, 19 in PH, 10 in VE and three in PI, were not found, likely owing to the imperfect draft genome assembly state of wheat genome [52]. The promoter sequences for three genes in barley, two in PH and one in PI, were removed because each of them only contains 10 nucleotides (excluding sequence gaps). The promoter sequences for all 60 Brachypodium genes were found.

**Table 1:** Numbers of homologous genes and promoters in photoperiod (PH), vernalization (VE), and pathway integration (PI) in Arabidopsis, Brachypodium, wheat and barley. Numbers in the parentheses indicate average promoter sequence length

	Arabidopsis		Brachypodium		Wheat		Barley	
	Gene	Promoter	Gene	Promoter	Gene	Promoter	Gene	Promoter
PH	40	39 (710)	34	34 (930)	124	105 (882)	40	38 (868)
VE	21	20 (905)	21	21 (1000)	50	40 (843)	14	14 (946)
PI	7	7 (1000)	5	5 (1000)	21	18 (827)	7	6 (820)
Total	68	66 (804)	60	60 (961)	195	163 (866)	61	58 (883)

### GC-content at predicted TFBSs

In anticipation of marked interspecific difference in GC content in the promoter regions, we created the species-specific background models for Arabidopsis, Brachypodium, wheat and barley (Additional file 1: Table S1), instead of the default background model of FIMO [28] for TFBS prediction. We predicted 1106, 1411, 1547, and 867 TFBSs in the promoter regions of the PH, VE and PI genes in Arabidopsis, Brachypodium, wheat and barley, respectively (Additional file 3). Due to the different lengths of the promoter sequences used in each species (Table 1), the TFBS density (number of TFBSs per KB sequences) differed among the four species: 21.9 (Arabidopsis), 25.3 (Brachypodium), 12.0 (wheat), and 17.8 (barley).

The estimated GC content in the entire promoter sequences was only about 32 % in Arabidopsis, and over 40 % in the three cereal species (Table 2), but all four species were GC-poor in the promoter regions with one exception (the GC-content was slightly over 50 % for the PI genes in Brachypodium). This result is in agreement with the general AT-rich feature of plant promoter sequences [32, 33]. When focusing just on th predicted TFBS sequences, a similar GC-poor trend was found across the four species, with the GC content being slightly higher at the TFBS sequences than in the entire promoter sequences in Arabidopsis, wheat and barley, but slightly lower in Brachypodium. In contrast, the GC-content in the 5’ UTR region and coding sequence (CDS) region was higher than that in the TFBS sequences and intron and 3’ UTR. In fact, the 5’ UTR and CDS regions were found GC-rich (>50 %) for some pathways. There was more variation (greater standard deviation) in the GC-content in the TFBS sequences than other greions particularly in introns.

**Table 2:** The average GC percentages (± standard deviations) in different genomic regions of flowering genes in the three pathways of photoperiod (PH), vernalization (VE) and pathway integration (PI) in Arabidopsis, Brachypodium, wheat and barley. Abbreviations: TFBS: transcription factor binding site; CDS, coding sequence; UTR, untranslated region; NA, not applicable (as only one 5’ UTR sequence was found in PI genes of Brachypodium)

		Promoter	TFBS	5’ UTR	CDS	Intron	3’ UTR
Arabidopsis	PH	33.4 ± 4.3	34.2 ± 8.7	36.7 ± 4.4	45.4 ± 3.3	31.7 ± 2.6	32.5 ± 3.9
	VE	31.3 ± 3.3	32.1 ± 7.0	39.0 ± 4.0	43.3 ± 2.1	31.4 ± 2.5	33.5 ± 4.0
	PI	30.0 ± 3.4	34.1 ± 11.6	34.4 ± 2.5	46.8 ± 3.8	28.9 ± 1.8	31.9 ± 4.5
Brachypodium	PH	48.9 ± 7.0	47.3 ± 9.5	57.4 ± 5.3	51.9 ± 9.6	38.4 ± 3.0	41.6 ± 3.9
	VE	46.9 ± 8.4	45.0 ± 7.8	62.1 ± 4.9	49.4 ± 6.1	38.8 ± 1.7	41.1 ± 3.2
	PI	50.0 ± 9.0	46.3 ± 14.2	50.3 ± NA	64.8 ± 7.8	41.2 ± 2.5	41.5 ± 3.9
Wheat	PH	41.8 ± 8.2	43.3 ± 11.6	48.6 ± 11.3	51.0 ± 6.9	38.9 ± 3.6	43.2 ± 4.3
	VE	43.8 ± 7.7	46.4 ± 13.7	46.0 ± 12.2	48.5 ± 7.0	39.4 ± 4.7	39.4 ± 5.8
	PI	43.0 ± 11.0	47.4 ± 18.6	50.8 ± 9.9	61.3 ± 9.0	41.6 ± 6.7	41.4 ± 5.1
Barley	PH	42.9 ± 7.8	47.4 ± 12.6	54.8 ± 10.7	52.5 ± 7.3	38.9 ± 4.6	42.7 ± 5.4
	VE	39.9 ± 5.0	40.8 ± 14.4	46.6 ± 10.6	51.1 ± 6.0	37.8 ± 2.2	39.2 ± 6.3
	PI	45.3 ± 8.5	42.5 ± 21.7	46.3 ± 10.5	62.8 ± 9.0	43.3 ± 2.7	44.3 ± 8.6

### TFBS distribution

Among the different TF families, MADS-box TFs (see Additional file 3 for description of different TF families) had the highest total number and the highest number of putative TFBSs per gene in all four species with the total of TFBSs being 469 (Arabidopsis), 513 (Brachypodium), 673 (wheat), and 251 (barley) as given in Table 3. Similar high frequency was observed for the CSD (cold shock domain) TF family, with the number of TFBSs being 374 (Arabidopsis), 148 (Brachypodium), 308 (wheat), and 115 (barley). Other TF families showed differences in the predicted TFBSs between Arabidopsis and the three cereal species. For example, the bZIP and bHLH families had many predicted TFBSs in Arabidopsis but very few in the cereals. It should be noted that the numbers of putative TFBSs did not appear to depend on the numbers of PWMs for each TF family examined in this study. The MYB/SANT family, for example, had 52 PWMs, but only 25 TFBSs predicted in Arabidopsis, 33 in Brachypodium, and 17 in wheat and 11 in barley. In contrast, the MADS-box family had 12 PWMs and CSD had only one, but they both had numerous TFBSs as described above.

**Table 3:** Numbers of putative transcription factor binding sites (TFBSs) and genes for the major transcription factor (TF) families in Arabidopsis, Brachypodium, wheat and barley. The TF families were sorted in descending order of the number of PWMs used in the TFBS prediction

TF family^a^	No. PWMs	No. TFBSs (genes)			
		Arabidopsis	Brachypodium	Wheat	Barley
Myb/SANT	52	25 (15)	14 (8)	17 (15)	11 (10)
AP2	49	6 (5)	231 (28)	90 (44)	125 (33)
bHLH	30	39 (26)	1 (1)	10 (6)	17 (14)
bZIP	28	74 (43)	8 (6)	24 (21)	17 (13)
WRKY	27	3 (3)	0 (0)	2 (2)	1 (1)
HB	26	3 (3)	7 (7)	0 (0)	3 (3)
TCP	18	11 (8)	34 (17)	92 (37)	44 (16)
GATA	15	1 (1)	0 (0)	3 (2)	0 (0)
NAC/NAM	15	1 (1)	0 (0)	0 (0)	3 (3)
SBP	15	8 (8)	1 (1)	0 (0)	3 (3)
Dof	14	4 (4)	1 (1)	5 (5)	3 (3)
MADS box	12	469 (61)	513 (57)	673 (131)	251 (52)
AT hook	11	1 (0)	18 (14)	192 (100)	40 (23)
C2H2 ZF	11	0 (0)	3 (3)	0 (0)	13 (11)
CSD	1	374 (57)	76 (27)	308 (105)	115 (40)

^a^The full names of TF families are given in the “TF family names” tab of Additional file 3

A brief functional annotation of these TF families is described below: Myb/SANT- Secondary metabolism, cellular morphogenesis, signal transduction in plant growth, abiotic and biotic stress responses, circadian rhythm, and dorsoventrality; AP2- Flower development, cell proliferation, secondary metabolism, abiotic and biotic stress responses, ABA response, and ethylene response; bHLH- Anthocyanin biosynthesis, light response, flower development and abiotic stress; bZIP- Seed-storage gene expression, photomorphogenesis, leaf development, flower development defense response, ABA response, and gibberellin biosynthesis; WRKY- Defense response HB (Homeodomain)- Development (leaf, root, internode, and ovule), stem cell identity, cell differentiation, growth responses, anthocyanin accumulation, and cell death; TCP- Flower development, asymmetry; GATA- Light response; NAC/NAM -Development, pattern formation, and organ separation; SBP- Plant development; Dof- Seed germination, endosperm-specific expression, and carbon metabolism; MADS box- Flower development, fruit development, flowering time, and root development; AT hook- Plant organ size and yield; CSD- Freezing tolerance, embryo development, flowering time, and fruit development; C2H2 ZF- Flower development, flowering time, seed development, and root nodule development

It is also evident from Table 3 that the numbers of flowering genes used to predict TFBSs for different TF families showed some interesting contrasts between Arabidopsis and the three cereal species. For example, for the MADS-box TFs, the percentages of flowering genes used to predict TFBSs were high across all the species: 61, or 92 % of total genes examined in Arabidopsis, 57 or 95 % in Brachypodium, 131 or 88 % in wheat, and 52 or 88 % in barley. On the other hand, for the AP2 family, which is referred to as the AP2/EREBP (ethylene-responsive element binding protein), the percentages of flowering genes used for TFBS prediction varied considerably among the species: only eight genes or 12 % of total genes examined in Arabidopsis, 54 or 90 % in Brachypodium, 46 or 31 %, in wheat, and 33 or 56 % in barley. Overall, there is little relationship between the number of genes used for TFBS prediction and the size of a TF family in individual species. Most noticeably, while NAC and WRKY are large TF families in Arabidopsis [53], the number of genes were limited.

There were overlaps between some predicted TFBSs for the same or different TF families within the same promoter region of a given gene. The average numbers of average of TFBSs were: 12 or 68 % of total predicted TFBSs in Arabidopsis, 15 or 63 % in Brachypodium, 6 or 58 % in wheat, and 10 or 62 % in barley. The overlapping occurred in six different TF families with five in Arabidopsis. Additionally, these TFBS overlaps occur most frequently in the MADS box and CSD TF families (Additional file 4).

### Relationships between TFBS numbers and gene expression profiles

**Fig. 1:**
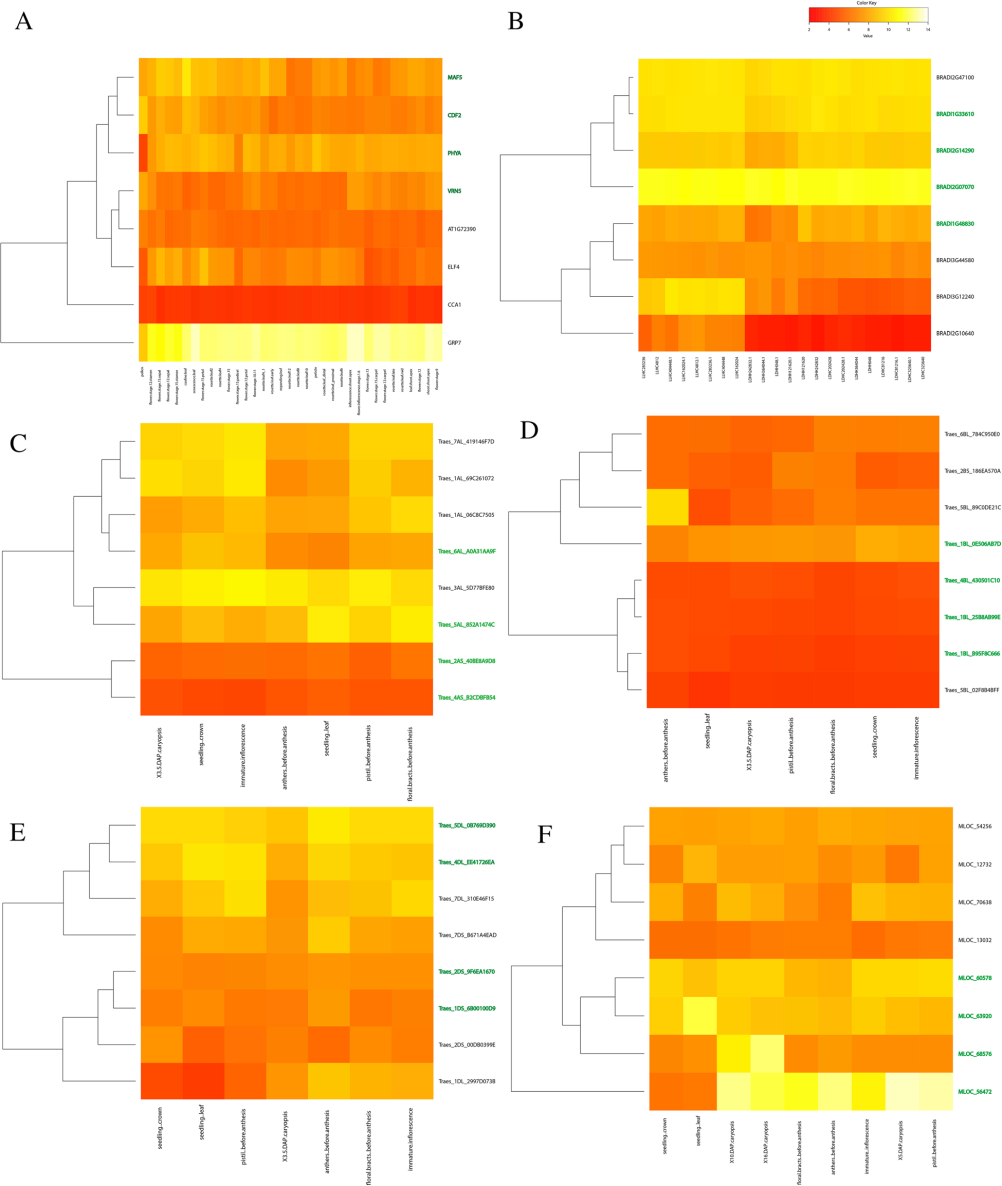
The expression profiles of the four genes each with the least (in black) and most (green) predicted transcription factor binding sites (TFBSs) in Arabidopsis (**a**), Brachypodium (**b**), wheat (**c**, **d**, and **e** for wheat A, B, D genomes), and barley (**f**)

Our analysis focused on four genes with the least or most numbers of predicted TFBSs in Arabidopsis, Brachypodium, wheat (with A, B, and D genomes being treated separately) and barley as summarized in Additional file 1: Table S2. This analysis assessed whether the genes with similar numbers of predicted TFBSs tend to exhibit similar expression patterns. The genes with no microarray expression data available in PLEXdb were excluded. Figure 1 showed the expression profiles of the four genes with the minimum and maximum numbers of TFBSs for MADS-box and CSD TF families. Generally, the genes with similar TFBS numbers for MADS and CSD TFs showed similar expression patterns across the tissues analyzed. This is particularly true in Arabidopsis, Brachypodium, wheat B genome and barley (Fig. 1A, B, D, F). The situation was different in wheat A and D genomes, with less consistent patterns for genes with similar TFBS profiles (Fig. 1 C, E).

### Intraspecific and interspecific TFBS divergence

**Fig. 2:**
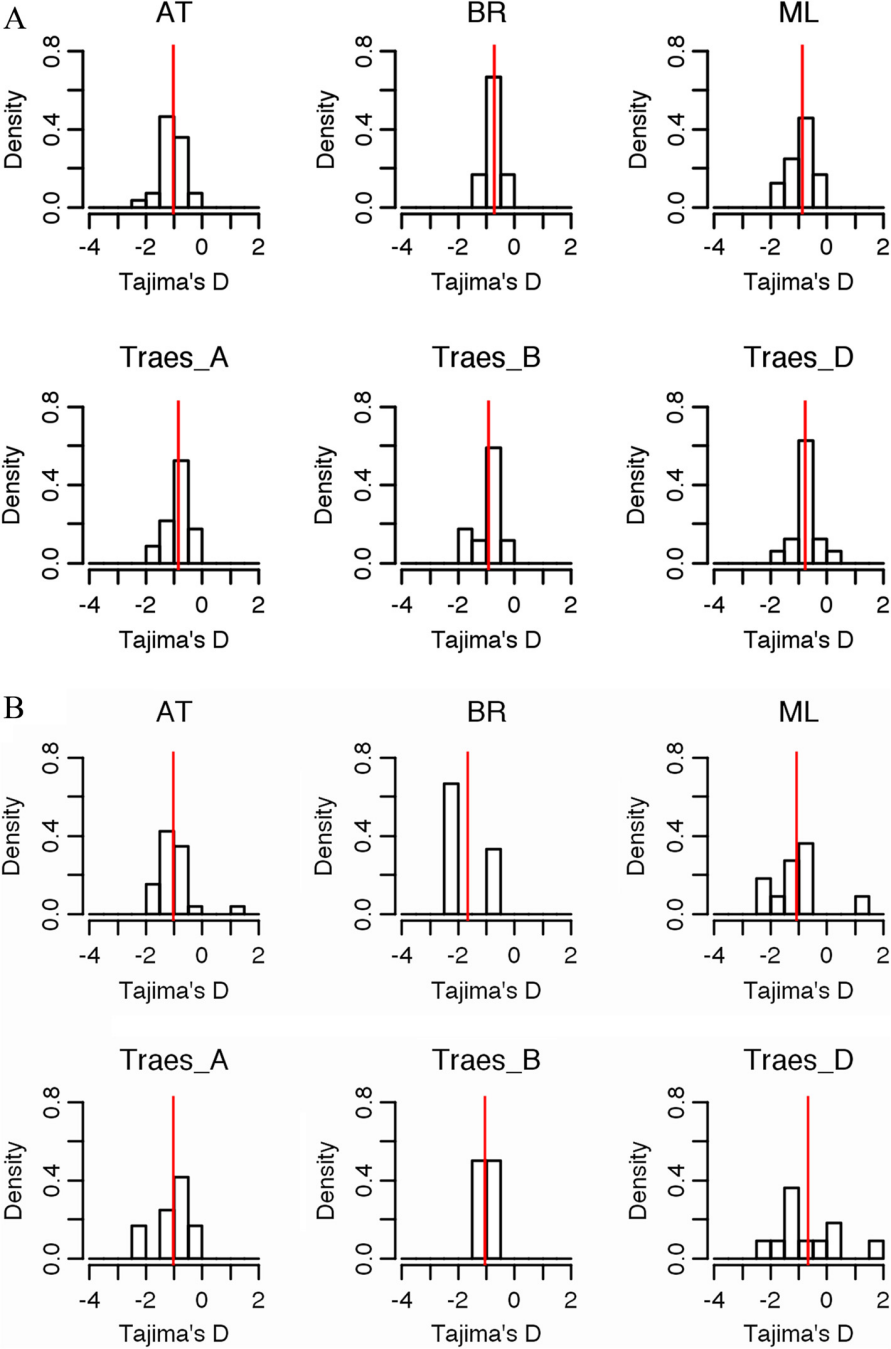
The histograms of Tajima’s *D* values of the binding sites for MADS-box (**a**) and CSD (**b**) transcription factor families within Arabidopsis (AT), Brachypodium (BR), barley (ML), and wheat (three genomes A, B, and D being treated separately). The red line in each histogram indicates the mean *D* value

Our within-species TFBS motif diversity estimation focused only on the two TF families: MADS-box and CSD (Additional file 5), where the predicted TFBSs were numerous enough for diversity and divergence assessment in the individual species. The distribution of their divergence was shown in Fig. 2. The average *D* for MADS TFBSs was -1.03 (ranging from -2.37 to -0.03) in Arabidopsis, -0.73 (-1.30 to -0.24) in Brachypodium, -0.85 (A genome; -1.88 to -0.23), -0.93 (B genome; -2.0 to -0.07), -0.77 (D genome; -1.56 to 0.29) in allohexaploid wheat, and -0.89 (-1.84 to -0.21) in barley (Fig. 2A). These results indicate the range of TFBS specificity levels for MADS-box TFs from the highest in Arabidopsis to the lowest in Brachypodium. In contrast, the TFBS specificity level for the CSD TF family was the highest in Brachypodium with the average *D* being -1.67 (-2.01 to -0.97) in Brachypodium, -1.02 (-1.76 to 1.17) in Arabidopsis, -1.04 (A genome; -2.01 to -0.26), -1.05 (B genome; -1.3 to -0.77), -0.67 (D genome; -2.01 to 1.63) in wheat, and -1.08 (-2.36 to 1.22) in barley (Fig. 2B). The specificity of MADS and CSD TFBSs in Arabidopsis was roughly at the same level. In barley, MADS TFBSs were more divergent than CSD TFBSs. In the wheat, MADS TFBSs appeared to be more divergent than CSD in A and B genomes but not in the D genome.

The interspecific diversity analysis indicated that the average *D* was -0.80 (-1.62 to 1.45) for TFBS, -1.08 (-1.51 to -0.36) for coding sequences (CDS), and -0.93 (-1.54 to 0.08) for introns (Additional file 1: Figure S1). On average, the observed interspecific divergence for TFBSs exceeded that of coding sequences. The relative conservation of CDS across these species was expected, as *CO* (AT5G15840) and *CONSTANS-LIKE 2* (*COL2*, AT3G02380 genes are involved in the critical node (circadian rhythm) of the photoperiod pathway. However, the TFBSs had a large range of Tajima’s *D*, suggesting that some of them would be more divergent than others. It is of interest to note that the divergence of introns of these genes between these species as judged from its *D* value lied intermediate between the estimates of the divergence for TFBS and CDS (Additional file 6).

**Fig. 3:**
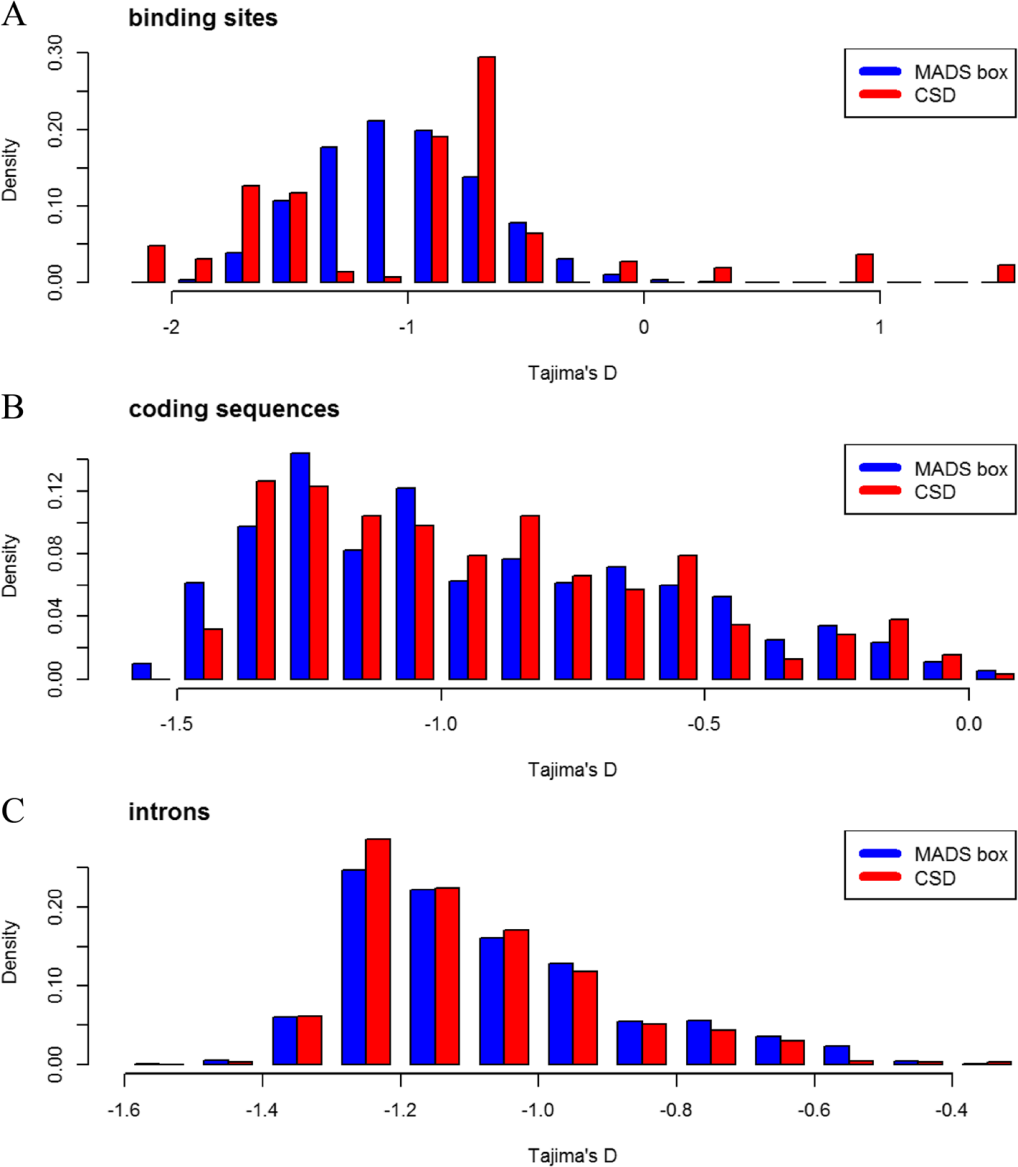
The Tajima’s *D* values of the binding sites (**a**), coding sequences (**b**), and introns (**c**) for the MADS box and CSD (cold shock domain) transcription factor families across genomes of Arabidopsis, Brachypodium, barley, and three genomes (A, B, and D) allohexaploid wheat

Our analysis indicated that TFBSs for MADS-box were more conserved than CSD among the four species (Fig. 3A). For different TF families, the average *D* of MADS TFBSs was -1.02 (-2.11 to 1.06), whereas it was -0.94 (-2.02 to 1.45). The intron sequences of the genes with MADS-box and CSD TFBSs were also compared (Fig. 3B), and the divergence was roughly the same in MADS and CSD. This was also the case for the coding sequences (CDS) of genes with MADS and CSD TFBSs (Fig. 3C).

### TFBS mapping over the flowering pathways

**Fig. 4:**
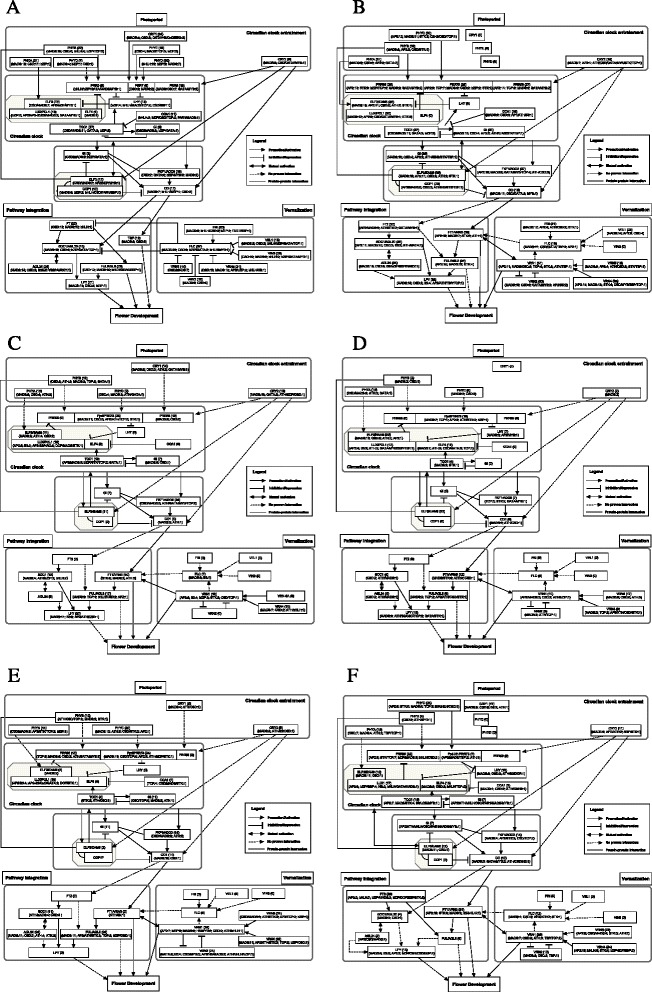
Numbers of predicted transcription factor binding sites (TFBSs) mapped onto the genes in the pathways of photoperiod, vernalization and pathway integration in Arabidopsis (panel **A**), Brachypodium (panel **B**), wheat genome A (panel **C**), wheat genome B (panel **D**), wheat genome D (panel **E**) and barley (panel **F**). The shadowed boxes within each image enclose the proteins that can act together in the pathway. The panel within each image shows different line symbols that represent different interactions between the proteins in the flowering pathway. Abbreviations: ATH, AT hook; CSD, cold shock domain; HB, homeodomain; STK, storekeeper; TBP, TATA-binding protein. A gene name followed with (0) indicates that no gene was found in this study. The gene names and their identifiers in each genome were given in Additional file 2

Since there are obvious differences in PH, VE and PI pathways between Arabidopsis and the three cereal species, many flowering genes were not shared between them. For example, in the photoreceptor component of PH pathway, there were a total of five genes for phytochromes [*PHYTOCHROME A (PHYA*, AT1G09570), *PHYTOCHROME B* (*PHYB*, AT2G18790), *PHYTOCHROME C* (*PHYC*, AT5G35840), *PHYTOCHROME D* (*PHYD*, AT4G16250), *PHYTOCHROME E* (*PHYE*, AT4G18130], and two genes for cryptochromes [*CRYPTOCHROME 1* (*CRY1*, AT4G08920) and *CRYPTOCHROME 2* (*CRY2*, AT1G04400)], but *PHYC* was not predicted in Arabidopsis, nor did *PHYD* and *PHYE* in the three cereals and *CRY1* in Brachypodium, wheat B genome and barley (Fig. 4). To help bridge the difference in the flowering ways between Arabidopsis and the three cereal species, we proposed new hypothetical links (dashed arrows in Fig. 4B - F) between several genes, including putative *SUPPRESSOR OF OVEREXPRESSION OF CO 1* (*SOC1*, AT2G45660) and *LEAFY* (*LFY*, AT5G61850) in the three cereal species. While these proposed links need to be confirmed in future experimental studies, our results showed that many SOC1 (a MADS-box TF) binding sites were predicted in the upstream of *LFY*, suggesting their regulatory relationship. For example, 12 SOC1 binding sites were predicted in the promoter of *BdLFY* (BRADI5G20340); 10 and three binding motifs were found for *LFY-A* (Traes_2AL_83D0D0C3F) and *LFY-B* (Traes_2BL_8DEC0EFBF). In the barley *HvLFY* (MLOC_14305), three SOC1 binding motifs were predicted (Additional file 3).

For those genes found in the flowering pathways, the number of TFBSs of the same genes varied considerably among the species (Fig. 4). In all four species, the predicted TFBSs were most frequent for the MADS-box and CSD family TFs that regulate the photoreceptor genes in the phtoperiod pathway. However, there were exceptions, e.g. *PHYD* in the Arabidopsis wherethe TFBSs for the bHLH and bZIP family TFs were most frequent. another noticveable difference between the Arabidopsis and cereals is that the AP2 TFs were frequent participants of DNA binding in the cereals particularly in Brachypodium but almost absent in the Arabidopsis. Similar patterns of TFBS distributions were found in the vernalization pathway with the predicted TFBSs for MADS-box and CSD family TFs being most frequent across all the species. it appeared that the cereal *VRN1* (homologous to *APETALA 1* (*AP1*)/*CAULIFLOWER* (*CAL*)/*FRUITFULL* (*FUL*), not *VRN1* in Arabidopsis) and *VRN4* (no Arabidopsis equivalent found in Arabidopsis), AP2 family TFs were important contributors to DNA binding for vernalization regulation, judging from the frequencies of their TFBSs we predicted. For the pathway integrator genes such as *FLOWERING LOCUS T* (*FT)*, *SOC1*, *AGAMOUS-LIKE 24* (*AGL24*), *FUL* and *LFY*, the patterns similar to those found in the photoperiod and vernalization pathways appeared again: the most frequent occurrence of TFBSs for MADS-box and CSD family TFs in Arabidopsis and cereals, but the equal frequent occurrence of TFBSs for AP2 family TFs in the cereals only.. For example, of the 23 TFBSs predicted for the *FT* gene in Arabidopsis, 12 (52 %) were for CSD TFs and 10 (43 %) were for MADS-box TFs. In the three cereal species, on the other hand, while MADS-box (and to a lesser extent, CSD) remained to be the main TF families for the predicted TFBSs, AP2 become another major TF family for regulating the pathway integration particularly in Brachypodium and barley. For example, of the 30 TFBSs predicted in Brachypodium for the *FT1*gene, 16 (or 53 %) were for AP2 TFs, seven (23 %) for MADS-box TFs and the rest were for the STK and ATH TFs.

## Discussion

In this work, we predicted TFBSs in the promoter regions of flowering genes involved in the PH, VE and PI pathways in two model plants, Arabidopsis and Brachypodium, and two important cereal crops, wheat and barley. We chose the two major flowering pathways (PH and VE) and their integrator (PI) for TFBS prediction because these pathways are regulated by the well-known gene regulatory networks [3, 6, 54], but little experimental evidence is available for characterizing how *cis*- regulatory elements (CREs) in the promoter regions of the genes are interacted with each other to activate or repress the expression of a network of genes along the pathways. For easy identification and comarison within and among the four species, we mapped the predicted TFBSs onto appropriate flowering genes present in the pathways (Fig. 4). In this fahsion, a gene regulatory network is readily formed to visualize the flow of gene regulation within the network (i.e., how one gene regulator is controlled by another in the network of genes within and between the pathways). To the best of our knowledge, this is the first attempt to link the pathways with predicted TFBSs, thereby providing an opportunity for pathway-guided prediction of TFs for specific genes in future studies.

Recent sequencing of several genomes of non-model plants including the large and complex genomes of wheat and barley [52, 55, 56] has allowed for prediction or identification of important genes (e.g., flowering genes) in the crop species based on well-annotated genes in model species such as Arabidopsis or Brachypodium. However, these gene annotation data can only be more effectively utilized if more is learned from patterns and properties of CREs. In our study, we focused on predicting distributions and patterns of TFBSs in the promoter regions of flowering genes. While the promoters are essential for transcription regulation of flowering or other functional genes across the genome, they alone can only produce basal levels of mRNA. Additionally, TFBSs in promoter regions often bind to a set of widely used and highly conserved TFs and thus they are not the major cause of *cis*-regulatory divergence among different species. In contrast, enhancers often turn on the promoters at specific genomic locations, times, and levels, and that is why they are sometimes known as the “promoters of the promoter.” Enhancers exhibit more interspecific variability and thus they are more often considered to be responsible for *cis*-regulatory divergence. However, unlike promoters, enhancers are more difficult to be located because they appear to be in upstream (5’), downstream (3’) or in the intron(s) of the gene they regulate; they can also be located far away from the gene.

Recently, a new open chromatin signature-based enhancer prediction system was developed for enhancer identification in Arabidopsis and other plants [57] Genome-wide patterns and distributions of other types of CREs such as silencers and insulators remain poorly understood. Thus, future studies can identify reproducible sequence patterns and genomic locations of TFBSs for enhancers and other less well-characterized CREs for bioinformatic predictions similar to what we did for promoters in this study.

While TFBS prediction is an important first step towards molecular characterization of gene regulation in plant species, it remains more difficult than gene prediction for the following reasons. First, regulatory regions control the transcription of genes but do not directly code for an identifiable product or function. Thus, TFBSs must be predicted from DNA sequences alone. Second, TFBSs are typically short in length, ranging from 5 to 31 nucleotides with an average length of merely 10 bp in eukaryotes [58]. Hence, it can be difficult to predict TFBSs using simple sequence analysis tools such as BLAST. Third, most TFBSs are highly degenerate [59, 60], which are reflected in the different probabilities of the four nucleotides at each position of PWMs. Consequently, a similar promoter sequence can be recognized by different groups of TFs and a TF may bind to more than one motif [61]. To further improve the quality of the predicted TFBSs, we implemented several filtering steps for both input and output files. First, those promoter sequences with large gaps (N’s). were removed from the input file. Second, high-quality PWMs (preferably derived from direct experimental data) were used for TFBS prediction. PWMs from public databases, such as JASPAR, TRANSFAC and CIS-BP [24, 30, 31], might be redundant. For example, even in the same database, identical TFs may sometimes be represented by different matrices that are obtained with different methods [62]. Consequently, redundant or very similar PWMs were removed to reduce the false positive rate. Third, for the FIMO output files, the predicted motifs were examined to only retain the most significant TFBSs or the longest motif covering short ones within the same TF family.

The largest numbers of TFBSs were predicted for MADS-box and CSD TF families, suggesting their important roles in flowering regulation. Several reasons for such TFBS motif abundance in MADS-box and CSD TF families may be speculated. First of all, the large number of hits may be due to the possibility that the PWMs for these TF families are less conservative. To check out this possibility, we calculated the Kullback–Leibler divergence (DKL) indices [63] for all 371 PWMs, corresponding to 345 cognate TFs. The DKL values had a wide range from -0.2179 to -6.5575. A DKL index should be close to zero if the letter (basepair) distribution is close to a uniform distribution (i.e., p- > 0.25); otherwise it would be far from zero. According to this criterion, the MADS-CSD motifs are actually more conservative as their DKL values were < -4.0. So the large number of hits in TFBS motif search is not necessarily caused by the less conservation of PWMs for the MADS-CSD motifs.

There are other possible reasons for the TFBS motif abundance in MADS-box and CSD TF families as well. The roles of MADS-box TFs in flowering control have long been established, and CSD TFs are mainly involved in cold acclimation but some of them are also related to flowering time [64–70]. Furthermore, most MADS-box and CSD motifs overlap in the promoter region of a target gene, suggesting that they might play cooperative functions in the regulation of photoperiod and vernalization responses. Additionally, we found genes with similar number of MADS-box and CSD TFBSs often show similar expression (coexpression) patterns in different tissues and developmental stages. It is somewhat surprising that we predicted the highest density of putative TFBSs in Brachypodium. This might be at least partially due to the larger number (195) of PWMs derived from monocot species than the number (176) of PWMs from Arabidopsis. Another reason might be the higher quality of promoter sequences in Brachypodium than in wheat and barley. The functional implication of higher TFBS density in Brachypodium may be a topic for further research.

It should be noted that only a limited number of PWMs are available in the two monocot crop species in our study, wheat and barley, because few functional genes such as those related to flowering are molecularly characterized in these large, complex genomes. The TFBS prediction based on very few PWMs would be unreliable. For this reason, maize, along a few other species, was added to the list of the monocot species in our initial compiling of PWMs. In particular, maize had 209 PWMs compared to nine PWMs for wheat and six PWMs for barley. Brachypodium is phylogenetically closer to wheat and barley than maize and had a similar number (192) of PWMs to maize, but it is a wild species with a potentially large number of ancient or doimestication genes that have been eliminated or modified from the geneomes of maize, wheat and barley during their domestication and selective breeding [71]. A check based on the DKL index [63] shows a similarity between motifs of maize, wheat and barley. Thus adding maize to the list would have helped to improve the accuracy of the TFBS prediction through borrawing the PWM information from the monocot species such as maize with close phylogeny and similar demostication levels.

The superimposition of TFBS numbers of the major TF families in the flowering pathways allows us to compare the TFBS profiles in promoters of the regulated genes for PH, VE and PI pathways in the four species. For the orthologous genes we determined among the four these species, both the similarity and differences were found. For example, TFBSs of MADS-box TFs were predicted for the orthologous photoperiod gene *CO* in the four species. On the other hand, the floral integrator *FT* gene showed different TFBS profiles. For example, the Arabidopsis *FT* gene was predicted to be regulated by CSD and MADS-box TFs. However, both the Brachypodium *FT* (BRADI1G48830) and barley *FT* gene (*VRN3-H*, MLOC_68576) appeared to be regulated by TFs in AP2, MADS and STK (storekeeper) families. The putative wheat *FT1-A* (Traes_7AS_EBD5F1F54) might be controlled by storekeeper (STK), MADS and AT hook (ATH) TF families, similar to *FT1-B* (Traes_7BS_581AA844D) and *FT1-D* (Traes_7DS_12C14942B, though only had one predicted TFBS each for ATH and STK family TFs). This difference of transcriptional regulation in *FT* genes may be supported by the view in [6]: the roles of *FT*-like genes appear to be highly conserved, but the TFs controlling their transcription vary during evolution, allowing transcription of *FT*-like genes in response to different conditions. Overall, the relative similarity of TFBS profiles in PH, VE and PI pathways are consistent with the conservation and divergence of these flowering pathways between the species [6, 8, 11].

Our GC content analysis was used as an indicator of promoters, because previous studies of regulatory sequences suggested that GC content has a significant difference between dicots and cereals, and methods of promoter identification based on DNA free energy profile were developed [32, 33]. Additionally, TFs from different families often prefer binding to regions with low or high GC content surrounding the core TFBS [72], thus it would be interesting to examine how the different GC content in the promoter regions of these genes might affect the binding environment of different TFs. In flower development, epigenetic regulation such as DNA methylation, histone modification, nucleosome positioning, and chromatin accessibility also plays an important role [21, 73–75]. Thus, it is possible that the different GC content in the promoter regions (particularly those surrounding predicted TFBSs) between Arabidopsis and cereal species might affect TF binding via epigenetic mechanisms.

The TFBS divergence relative to their corresponding coding sequences and introns was assessed using the Tajima test in VariScan [47, 48]. The Tajima’s *D* has previously been used to assess divergence patterns in the conifer EST (expressed sequence tag) data [76]. Our analysis suggests different binding preference of each TF family in each species, including MADS-box and CSD. And among these four species, the TFBSs diverged faster than their corresponding CDS, similar to the binding sites of Ste12 and Tec1 regulators in yeast [77]. More interestingly, introns might also diverge faster than CDS (but slower than TFBS). The potential function of introns in TFBSs and flowering regulation has gained growing attention [22, 23, 78, 79].

Despite its popularity for TFBS predictions, FIMO, like most motif search tools, may be prone to a high rate of false positives. There is definitely a need to further check the validity of FIMO-based predictions. We have tried out some recently-developed tools for comparative assessment with FIMO. In particular, BoBro 2.0 of Ma et al. [80] is an integreated toolkit aiming at improved control of false positive rate for the predicted TFBS motifs and higher prediction sensitivity through efficient handling of sequence variation in motifs. The preliminary results show that some TFBS motifs predicted by both FIMO and BoBro 2.0 have overlapping but not identical start/end positions while the majority of others are located at separate regions of the genome. Since BoBro 2.0 was developed initially for prokarotic genomes, further investigations are needed to make BoBro 2.0 or similar tools well adapted to the TFBS predictions for eukarotic genomes of higher plants such as those in our study.

Our FIMO-based TFBS motif search is based on known binding sites. When such knowledge is not available, de nevo motif discovery [81] has been suggested as an alternative approach to predicting TFBS motifs. In the de nevo motif discovery, multiple sequences are input to detect one or more candidate motifs. However, while the use of de novo motif search could have allowed us to find many more motifs, it would have been difficult to associate the motifs discovered de novo with known TFs. Without knowledge of their potential TFs, the predicted motifs would be of very limited value. Even though the de nevo motif search was not used in our study, it certainly needs to be explored in future studies.

## Conclusions

Using the FIMO motif discovery tool in MEMPpE [28], we predicted a large number of putative TFBSs in the promoters of the genes related to the PH, VE and PI pathways in Arabidopsis, Brachypodium, wheat and barley. The quality of the predicted TFBSs was improved through cleaning both the inputs (promoter sequences and PWMs) and the FIMO outputs. The genes with similar TFBS numbers tend to be co-expressed in different tissues of each species. Based on our intraspecific and interspecific Tajima *D*-statistics [47, 48], TFBSs from different TF families showed different divergence within each species, and TFBSs are more divergent compared with CDS and introns. The TFBS numbers for major TF families were superimposed in the flowering pathways with PathVisio and WikiPathways [50, 51], to show the similarity and difference between these four species. The TFBSs and TF-targeted gene associations presented in our study can be investigated for their roles in photoperiod and vernalization responses in the genomes of four plant species, especially in the large, poorly-characterized genomes of two cereal crops, wheat and barley.

## Additional files

Additional file 1: Table S1. Nucleotide frequency in promoter sequences of the whole genome used for background models in binding motif predictions in Arabidopsis, Brachypodium, Wheat and barley. Table S2. Summary of the ranges of numbers of transcription factor binding sites (TFBSs) of transcription factor (TF) families in Arabidopsis, Brachypodium, wheat and barley. Figure S1. The Tajima’s *D* of the binding sites (A), coding sequences (B), and introns (C) for all the analyzed transcription factor (TF) families across Arabidopsis, Brachypodium, barley, and wheat. (PDF 242 kb) Additional file 2: The ortholog groups and genes in the flowering pathways in Arabidopsis, Brachypodium, wheat and barley. (XLS 62 kb) Additional file 3: The predicted transcription factor binding sites (TFBSs) in the promoters of genes in the pathways of photoperiod, vernalization and pathway integration in Arabidopsis, Brachypodium, wheat and barley. (XLSX 406 kb) Additional file 4: The overlapping transcription factor binding sites (TFBSs) for each gene that is concurrent in Arabidopsis, Brachypodium, wheat and barley. (XLSX 197 kb) Additional file 5: The intraspecific Tajima’s *D* values of TFBSs in Arabidopsis, Brachypodium, wheat and barley. (XLS 110 kb) Additional file 6: The interspecific Tajima’s *D* values of TFBS, coding sequences (CDS) and introns. (XLS 1229 kb)
