# Supplementary material for: Bioinformatic prediction of transcription factor binding sites at promoter regions of genes for photoperiod and vernalization responses in model and temperate cereal plants
Source: BMC Genomics. 2016 Aug 8;17:573. doi: 10.1186/s12864-016-2916-7 (PMC4977670; doi:10.1186/s12864-016-2916-7)

Table S1. Nucleotide frequency in upstream sequences of the whole genome used for background models in binding motif predictions in Arabidopsis, Brachypodium, Wheat and barley. The upstream sequences of the genes in each species under this study were excluded. Values in parentheses are the nucleotide frequencies in each whole genome.

	Arabidopsis	Brachypodium	Wheat	Barley
A	0.3294 (0.3199)	0.2714 (0.2680)	0.2644 (0.2740)	0.2762 (0.2782)
C	0.1678 (0.1804)	0.2215 (0.2321)	0.2337 (0.2259)	0.2197 (0.2216)
G	0.1678 (0.1802)	0.2296 (0.2319)	0.2261 (0.2259)	0.2221 (0.2219)
T	0.3350 (0.3195)	0.2775 (0.2680)	0.2758 (0.2742)	0.2820 (0.2784)

Table S2. Summary of the ranges of numbers of transcription factor binding sites per gene by transcription factor (TF) families in Arabidopsis, Brachypodium, wheat and barley. CSD: cold shock domain.

	Arabidopsis	Brachypodium	Wheat A	Wheat B	Wheat D	Barley
CSD	1 - 16	1 - 12	1 - 11	1 - 6	1 - 14	1 - 9
MADS	1 - 19	1 - 30	1 - 11	1 - 14	1 - 14	1 - 21
Others	1 - 14	1 - 43	1 - 11	1 - 9	1 - 12	1 - 30
Total	1 - 36	1 - 47	1 - 27	1 - 25	1 - 30	3 - 34

Figure S1. The Tajima's *D* of the binding sites (A), coding sequences (B), and introns (C) for all analyzed transcription factor families across Arabidopsis, Brachypodium, barley, and wheat.

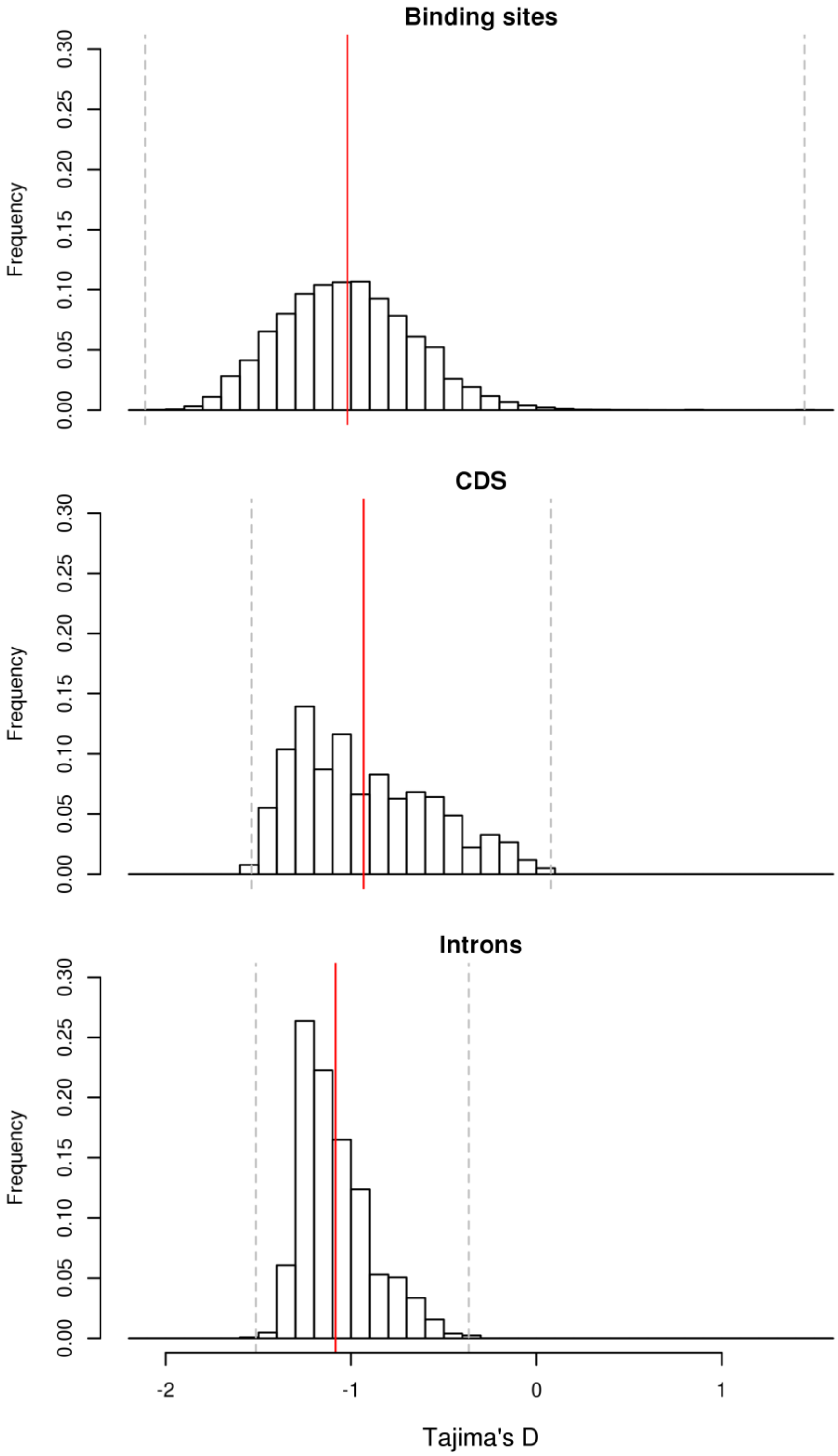

Supplement: Additional file 1: Table S1. — Nucleotide frequency in promoter sequences of the whole genome used for background models in binding motif predictions in Arabidopsis, Brachypodium, Wheat and barley. Table S2. Summary of the ranges of numbers of transcription factor binding sites (TFBSs) of transcription factor (TF) families in Arabidopsis, Brachypodium, wheat and barley. Figure S1. The Tajima’s D of the binding sites (A), coding sequences (B), and introns (C) for all the analyzed transcription factor (TF) families across Arabidopsis, Brachypodium, barley, and wheat. (PDF 242 kb) [file 12864_2016_2916_MOESM1_ESM.pdf]
